# Supplementary material for: Diallyl Disulfide Induces Chemosensitization to Sorafenib, Autophagy, and Cell Cycle Arrest and Inhibits Invasion in Hepatocellular Carcinoma
Source: Pharmaceutics. 2022 Nov 24;14(12):2582. doi: 10.3390/pharmaceutics14122582 (PMC9788602; doi:10.3390/pharmaceutics14122582)
Supplement: Supplementary file 1 [file pharmaceutics-14-02582-s001.zip › pharmaceutics-1964363-supplementary.pdf]

Supplementary material

Table S1 - Primes used for gene expression analysis by RT-qPCR

Primers ID	Symbol	Gene ID	Ref Seq ID
**H_ACTB_1**	*ACTB*	60	NM_001101
**H_CHECK_2**	*CHECK2*	11200	NM_10057352
**H_FOS_2**	*FOS2*	115116789	
**H_GAPDH_1**	*GAPDH*		NM_004964
**H_HPRT1_1**	*HPRT1*	3251	NM_000194
**H_MMP2_1**	*MMP2*	4313	NM_0011278913
**H_TNF_3**	*TNF*	7124	NM_000594

Table S2 - Antibodies used for the analysis of protein expression by western blot

Antibody	Host	Producer	CAT *number*
Anti-β-Actina	Rabbit	Cell Signaling Technology	8457S
Anti-LC3-II	Rabbit	Abclonal	A5618
Anti-Nrf2	Rabbit	Abclonal	A1244
Anti-IgG rabbit HRP-conjugate	Goat	Abclonal	AS014
